# Development and validation of a combined hypoxia- and metabolism-related prognostic signature to predict clinical prognosis and immunotherapy responses in clear cell renal cell carcinoma

**DOI:** 10.3389/fonc.2023.1162846

**Published:** 2023-11-10

**Authors:** Xin Wu, Wenjie Xie, Binbin Gong, Bin Fu, Weimin Chen, Libo Zhou, Lianmin Luo

**Affiliations:** Department of Urology, The First Affiliated Hospital of Nanchang University, Nanchang, Jiangxi, China

**Keywords:** clear cell renal cell carcinoma, hypoxia, metabolism, prognosis, immune

## Abstract

**Background:**

Hypoxia and metabolism are closely correlated with the progression of cancer. We aimed to construct a combined hypoxia- and metabolism-related genes (HMRGs) prognostic signature to predict survival and immunotherapy responses in patients with clear cell renal cell carcinoma (ccRCC).

**Methods:**

The RNA-seq profiles and clinical data of ccRCC were acquired from the TCGA and the ArrayExpress (E-MTAB-1980) databases. Least absolute shrinkage and selection operator (LASSO) and univariate and multivariate Cox regression analyses were applied to establish a prognostic signature. The E-MTAB-1980 cohort was selected for validation. The effectiveness and reliability of the signature were further evaluated by Kaplan–Meier (K-M) survival and time-dependent receiver operating characteristic (ROC) curves. Further analyses, including functional enrichment, ssGSEA algorithm, CIBERSORT algorithm, and expression of immune checkpoints, were explored to investigate immune status and immunotherapy responses.

**Results:**

We constructed a prognostic eight-gene signature with IRF6, TEK, PLCB2, ABCB1, TGFA, COL4A5, PLOD2, and TUBB6. Patients were divided into high-risk and low-risk groups based on the medium-risk score. The K-M analysis revealed that patients in the high-risk group had an apparently poor prognosis compared to those in the low-risk group in the TCGA (*p* < 0.001) and E-MTAB-1980 (*p* < 0.005). The area under ROC curve (AUC) of the prognostic signature was 0.8 at 1 year, 0.77 at 3 years, and 0.78 at 5 years in the TCGA, respectively, and was 0.82 at 1 year, 0.74 at 3 years, and 0.75 at 5 years in the E-MTAB-1980, respectively. Independent prognostic analysis confirmed the risk score as a separate prognostic factor in ccRCC patients (*p* < 0.001). The results of ssGSEA showed not only a high degree of immune cell infiltration but also high scores of immune-related functions in the high-risk group. The CIBERSORT analysis further confirmed that the abundance of immune cells was apparently different between the two risk groups. The risk score was significantly correlated with the expression of cytotoxic T lymphocyte-associated antigen-4 (CTLA4), lymphocyte-activation gene 3 (LAG3), and programmed cell death protein 1 (PD-1).

**Conclusion:**

The HMRGs signature could be used to predict clinical prognosis, evaluate the efficacy of immunotherapy, and guide personalized immunotherapy in ccRCC patients.

## Introduction

Renal cell carcinoma (RCC) is one of the most frequent genitourinary cancers, with an estimated 431,288 newly diagnosed cases and 179,368 deaths occurring in the world in 2020 according to global cancer statistics (1). Clear cell renal cell carcinoma (ccRCC) is the most prevalent subtype among RCCs, representing 75%–85% of all RCC cases (2). Most patients with localized ccRCC can be treated by partial nephrectomy or radical therapy, and overall clinical prognosis is satisfactory. Unfortunately, approximately 30% of patients with localized ccRCC will experience tumor recurrence or metastasis post-operation, which leads to poor prognosis (3). The most common treatment for advanced ccRCC is targeted therapy and immune checkpoint therapy, which significantly improved the prognosis of patients (4–6). However, owing to the high heterogeneity of cancer, some patients are unresponsive or resistant to targeted therapy or immune checkpoint therapy (7, 8), so that the treatment for patients with advanced ccRCC still represents a huge challenge. Thus, in the era of individualized cancer treatment, identifying reliable biomarkers are essential for optimizing risk stratification, providing individualized treatments, and predicting clinical prognosis (9, 10).

Hypoxia, a common and important feature of nearly all solid tumors, is caused by an inadequate oxygen supply due to the tumor uncontrolled growth beyond the existing vasculature (11). The tumor uncontrolled growth beyond the existing vasculature leads to hypoxia occurrence. It is well-known that hypoxia-inducible factors (HIFs) are the master transcription factors for oxygen homeostasis, which could drive the transcription of numerous genes that mediate invasion, metastasis, angiogenesis, epithelial–mesenchymal transition, immune evasion, and therapy resistance (12–14). Moreover, hypoxia mediates a cascade of metabolic reprogramming mostly via HIFs (15, 16).

Tumor metabolism is a hallmark of cancer and plays a significant role in tumor initiation and development. For cancer cells’ characteristic uncontrolled growth and proliferation, cancer cells change metabolic reprogramming to provide increasing energy and nutritional demands (17). Metabolic reprogramming change in TME could lead to immune escape of tumor cells and promote tumor growth (18). Alterations in metabolic reprogramming are closely related to cancer cell proliferation, migration, invasion, angiogenesis, drug resistance, and immune response (19–21). As the main hallmark in TME, both hypoxia and metabolism could influence the cancer cell tumor growth, metastasis and anti-tumor immune response. Hence, it is necessary to construct a robust prognostic model that combine hypoxia- and metabolism-related genes (HMRGs) signature in order to improve risk stratification and predict clinical outcomes for ccRCC.

To evaluate the clinical value of HMRGs in ccRCC, the bioinformatics analysis was performed by constructing an HMRGs prognostic signature in the TCGA and validating it in the E-MTAB-1980 database. Then, the effectiveness and reliability of the risk model were evaluated. In addition, we compared the difference of clinicopathological features, immune cell infiltration, and expression of common immune checkpoints between the high-risk group and low-risk group.

## Materials and methods

**Figure 1:**
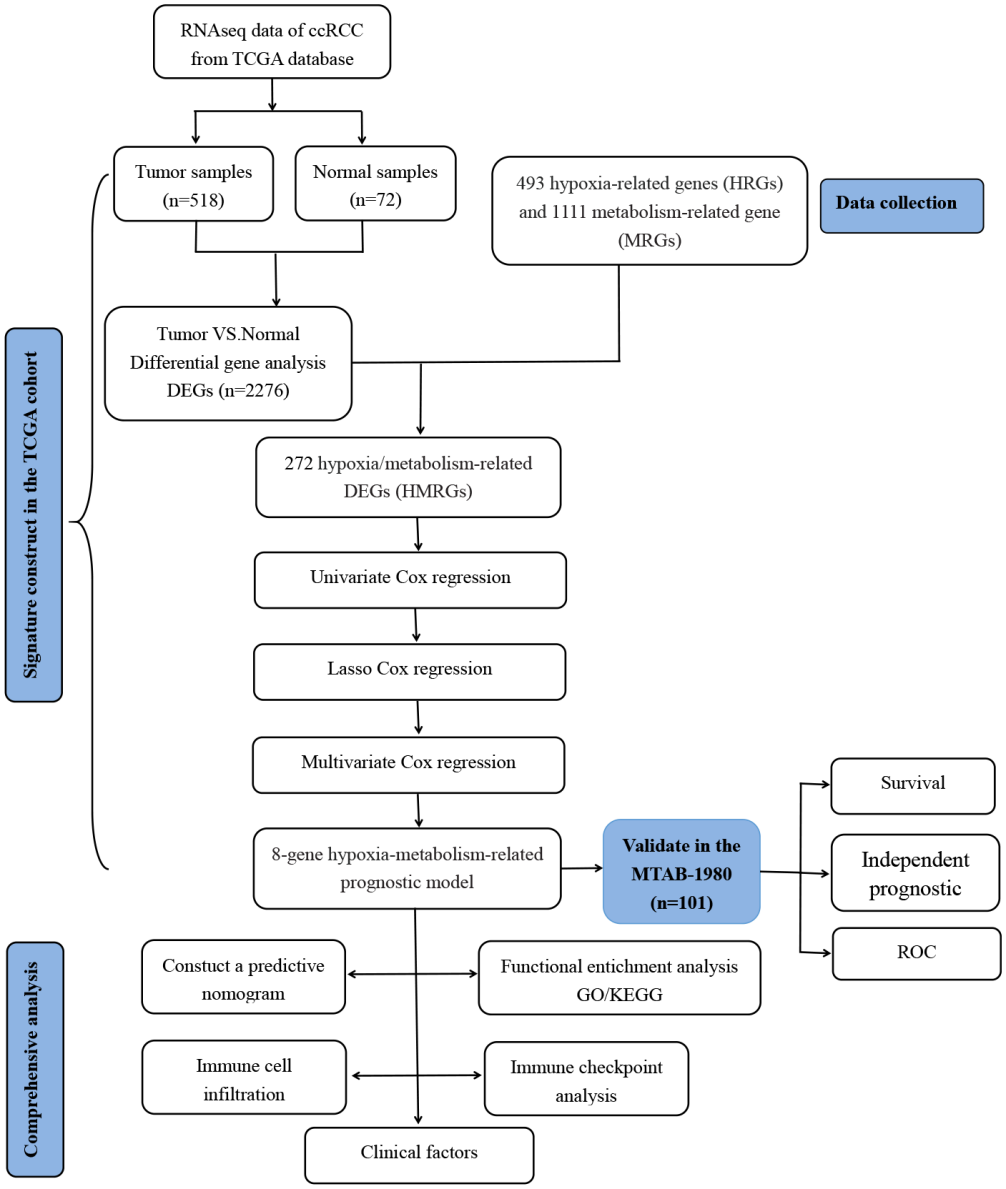
Flowchart of the analysis process in our study.

The flowchart of our study is presented in **Figure 1**.

### Data collection

For training cohort, RNA expression and clinical data of ccRCC cases were downloaded from TCGA (https://genomecancer.ucsc.edu). The E-MTAB-1980 dataset (https://www.ebi.ac.uk/arrayexpress/) was used for the validation cohort. Patients who survived less than 30 days were removed for subsequent analysis.

### Identification of hypoxia- and metabolism-related genes

In this study, 493 hypoxia-related genes and 1,111 metabolism-related genes were acquired from the Molecular Signatures Database (MSigDB; https://www.gsea-msigdb.org/gsea/msigdb/index.jsp) (**Supplementary Table 1**).

### Screening for HMRGs differentially expressed genes

Differentially expressed genes (DEGs) between tumor tissues and normal tissues were analyzed using the package “limma” according to |logFC| > 1 and adjusted *p* < 0.05. HMRGs were obtained through merging hypoxia-related genes and metabolism-related genes, and deduplication. The differentially expressed HMRGs were identified by intersecting DEGs with HMRGs, and visualizing them by the Venn diagram.

### Construction and validation of the prognostic signature based on HMRGs

Firstly, a univariate Cox regression analysis was conducted to identify the HMRGs associated with the prognosis in the TCGA. In addition, HMRGs with *p* < 0.05 were selected into LASSO regression to shrink the scope of prognosis genes’ screening. Subsequently, the candidate prognosis genes identified from LASSO analysis were selected into multivariate Cox regression analysis in order to assess their contribution as prognostic factors. Finally, a prognostic model was established using gene expression level and corresponding regression coefficients. The formula was as follows: risk score = β_mRNA1_ × Expression_mRNA1_ + β_mRNA2_ × Expression_mRNA2_ + β_mRNA3_ × Expression_mRNA3_ + …+ β_mRNAn_ × Expression_mRNAn_.

The patients were classified into the high-risk group and low-risk group based on the medium value of risk score. K-M survival analysis was conducted to compare the overall survival (OS) between the two risk groups. ROC curves were performed to evaluate the prediction efficiency of the risk model.

### Development and evaluation of a predictive nomogram

A predictive nomogram was constructed based on the independent prognostic factors, which included clinical parameters and HMRGs risk score, to predict the OS probability of ccRCC patients. Additionally, the predictive power of the nomogram was evaluated by ROC curves.

### Stratified analysis and comprehensive analysis of the prognostic model

To further evaluate the prognostic value of the HMRGs signature prognostic model, stratified analysis was performed in different subgroups based on clinical parameters. Next, the correlation between the risk score and clinicopathological parameters was investigated to better assess the role of the HMRGs signature prognostic model in the ccRCC development.

### GO and KEGG enrichment analysis

The package “limma” was conducted to analyze the DEGs between the high-risk group and low-risk group with the criterion set at |logFC| > 1 and adjusted *p* < 0.05. Then, Gene Ontology (GO) analysis and Kyoto Encyclopedia of Genes and Genomes (KEGG) pathway analysis were conducted by the package “ClusterProfiler” based on DEGs in order to explore the biological function of DEGs.

### Evaluation of tumor immune microenvironment

The ssGSEA algorithm was used to assess the relative abundance of immune cell infiltration between the high-risk group and low-risk group. Then, the CIBERSORT algorithm was applied to explore the infiltration levels of immune cell types. Furthermore, the expression of well-known immune checkpoint genes, including programmed cell death protein 1 (PD-1), programmed cell death protein ligand 1 (PD-L1), cytotoxic T lymphocyte-associated antigen-4 (CTLA4), and lymphocyte-activation gene 3 (LAG3), was compared between the high-risk group and the low-risk group.

### RNA extraction and quantitative real-time PCR analysis

Total RNA was extracted from five paired human ccRCC tissues and adjacent non-tumorous tissues using TRIzol Reagent (Invitrogen). The reverse transcription was conducted with PrimeScript™ RT reagent Kit (TaKaRa). Q-PCR was performed using SYBR green Premix Ex Taq II (Takara). GAPDH was selected as an internal control. The sequence of primers is shown in **Supplementary Table 2**.

### Statistical analysis

Statistical analyses and graphing were conducted using the R software (version R-4.1.2) or GraphPad Prism (version 8.0.2). The Student’s *t*-test was applied to compare the continuous data between two groups. Correlation coefficients were calculated using Spearman correlation analysis. *p-*value < 0.05 was considered as statistically significant. *p-*values were shown as follows: ns, not significant; *p < 0.05; **p < 0.01; ***p < 0.001.

## Results

### Screening of hypoxia- and metabolism-related prognostic genes in ccRCC in the TCGA cohort

**Figure 2:**
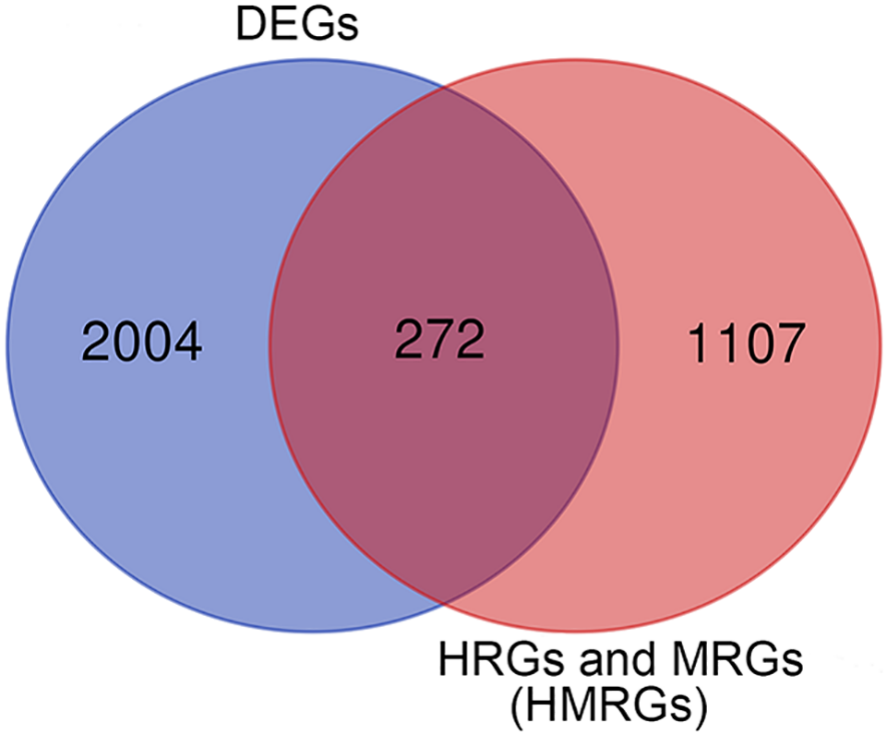
The differentially expressed HMRGs between normal and tumor tissue were identified by intersecting DEGs with HMRGs in the TCGA cohort.

In this study, according to the filtering criteria of the DEGs, 2,276 DEGs were obtained between ccRCC tissues and normal tissues. HMRGs were obtained through merging hypoxia-related genes and metabolism-related genes, and deduplication, and a total of 1,379 HMRGs were included. After intersecting DEGs with HMRGs, a total of 272 differentially expressed HMRGs were included (**Figure 2**). Then, a univariate Cox regression analysis was performed and a total number of 115 prognosis-related HMRGs were identified (**Supplementary Table 3**).

### Construction of the HMRGs prognostic signature in the TCGA cohort

The flowchart for the process of constructing the HMRGs prognostic signature is presented in **Supplementary Figure 1**.

**Figure 3:**
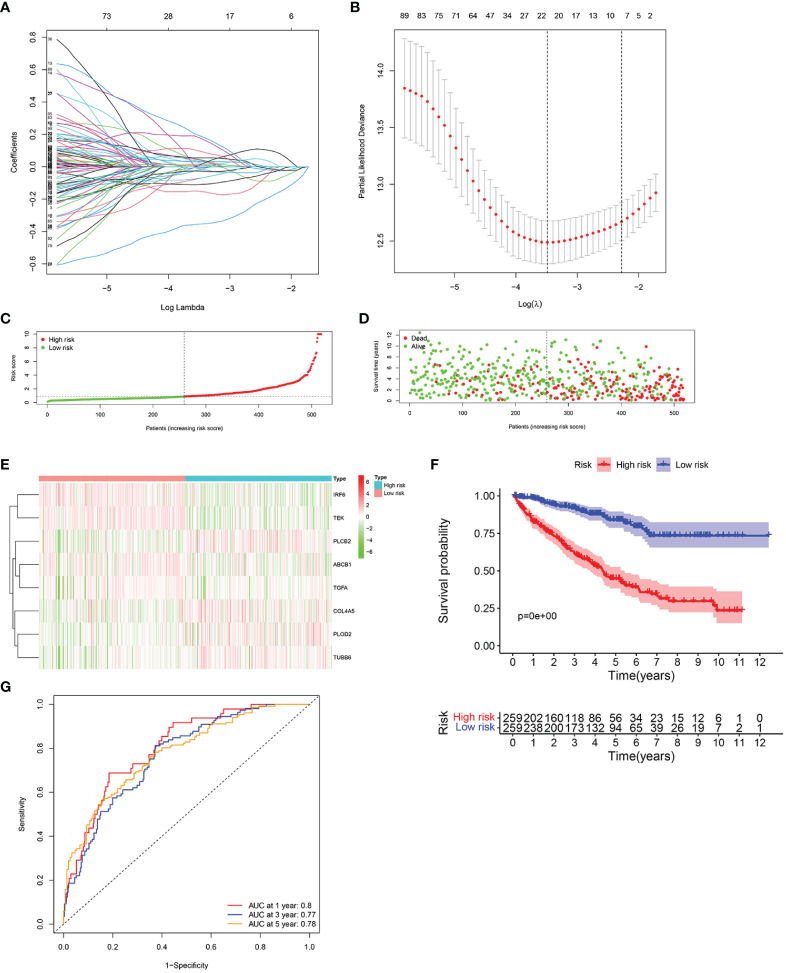
Construction of the HMRG prognostic signature in the TCGA cohort. **(A, B)** LASSO Cox regression analysis was performed to screen the key genes. **(C)** The median value and distribution of the risk score. **(D)** The distribution of survival status. **(E)** Expression of eight signature genes. **(F)** K-M curves for the OS. **(G)** ROC curve for 1, 3, and 5 years.

The LASSO regression analysis was performed to screen the key genes (**Figures 3A, B**). Next, the multivariate Cox regression was conducted to further filter out candidate genes in order to improve the prognostic ability of the model. Finally, eight genes, namely, IRF6, TEK, PLCB2, ABCB1, TGFA, COL4A5, PLOD2, and TUBB6, were selected to construct a prognostic signature. The risk score was calculated as follows: risk score = (−0.158 × the expression level of ABCB1) + (0.378 × the expression level of COL4A5) + (−0.151 × the expression level of IRF6) + (0.319 × the expression level of PLCB2) + (0.136 × the expression level of PLOD2) + (−0.406 × the expression level of TEK) + (−0.163 × the expression level of TGFA) + (0.394 × the expression level of TUBB6).

The patients were classified as high-risk group and low-risk group based on the median of risk score (**Figure 3C**). Patients in the high-risk group had a significantly higher mortality rate (**Figure 3D**). The expression of PLCB2, COL4A5, PLOD2, and TUBB6 was higher in the high-risk group, while the expression of IRF6, TEK, ABCB1, and TGFA were higher in the low-risk group (**Figure 3E**). Compared to normal tissues, the expression level of PLCB2, TGFA, PLOD2, and TUBB6 was significantly higher in ccRCC tissues, while the expression level of IRF6, TEK, ABCB1, and COL4A5 was significantly lower (**Supplementary Figures 2A–H**). We performed survival analysis based on the eight genes and found that patients with high expression of identified genes, such as PLCB2, COL4A5, PLOD2, and TUBB6, had a significantly poor survival, while patients with high expression of identified genes, such as IRF6, TEK, ABCB1, and TGFA, had a significantly favorable survival (**Supplementary Figures 3A–H**). K-M curves indicated that ccRCC samples with a low risk score had a significantly superior prognosis to those with a high risk score (*p* < 0.001) (**Figure 3F**). Moreover, the AUC reached 0.8 at 1 year, 0.77 at 3 years, and 0.78 at 5 years (**Figure 3G**). In general, these results suggested that the HMRGs signature model had a favorable efficacy in predicting the clinical prognosis.

### Validation of the prognostic signature in the E-MTAB-1980 cohort

To evaluate the reliability of the HMRGs signature model established from the TCGA cohort, risk scores were measured for the patients from the E-MTAB-1980 cohort with the same formula as the TCGA cohort (**Supplementary Figure 4A**). Similar to the TCGA cohort, patients in the high-risk group had a higher probability of death than patients in the low-risk group (**Supplementary Figure 4B**). The expression pattern of the risk model genes was similar to the TCGA cohort (**Supplementary Figure 4C**). K-M curves indicated that patients in the high-risk group had a significantly shorter OS than that in the low-risk group (*p* < 0.005) (**Supplementary Figure 4D**). In addition, the AUC of the HMRGs signature model was 0.82 at 1 year, 0.74 at 3 years, and 0.75 at 5 years (**Supplementary Figure 4E**), which exhibited a good prediction efficacy.

### Independence of the prognostic model and nomogram construction

**Figure 4:**
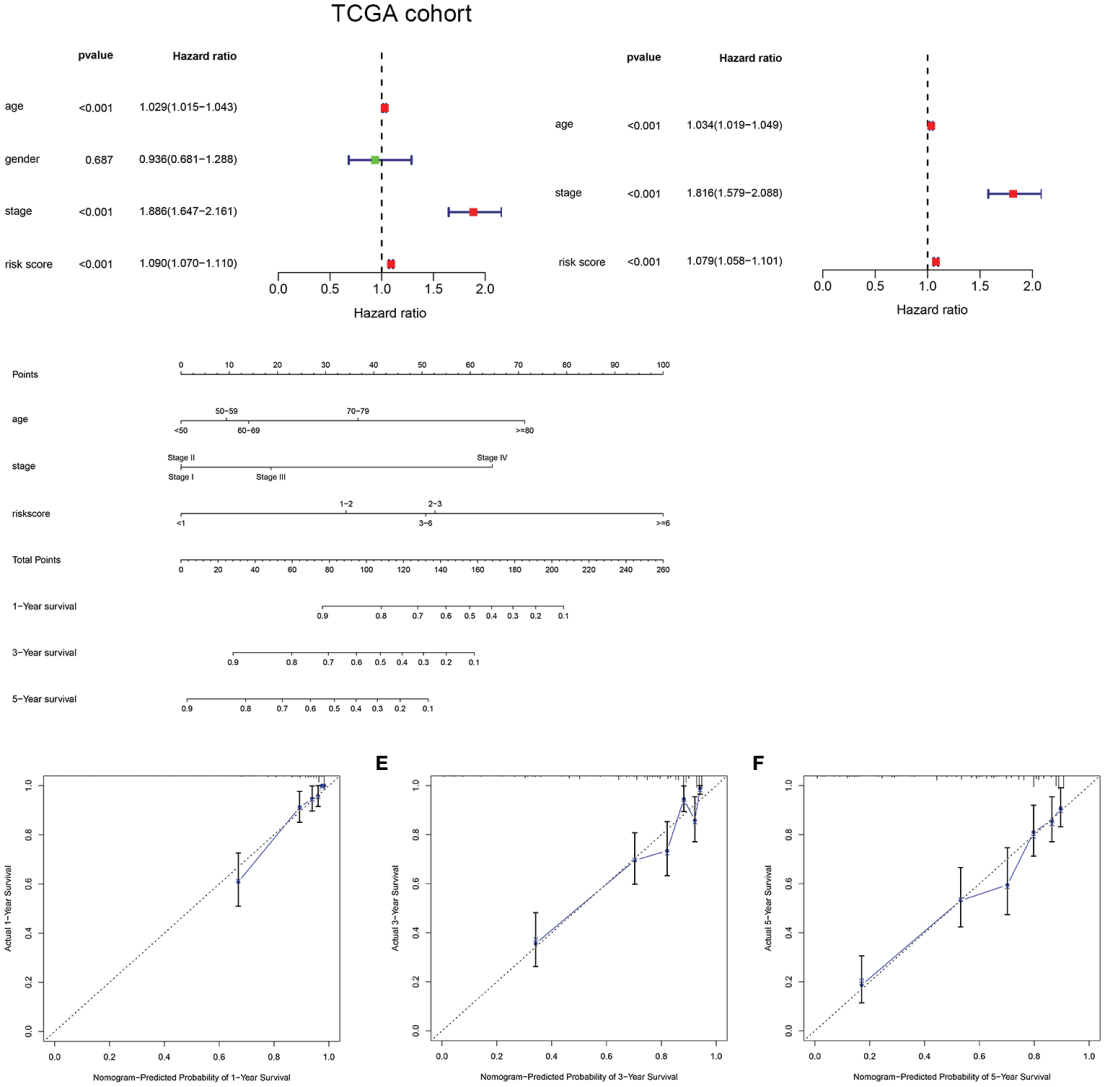
Development of a nomogram predicting OS in ccRCC. Univariate **(A)** and multivariate Cox regression **(B)** were performed in the TCGA cohort. Nomogram integrated age, stage, and risk score **(C)**. Calibration curve of the nomogram for predicting OS at 1, 3 and 5 years **(D**–**F)**.

To determine the independent prediction ability of the HMRGs signature model for clinical prognosis, univariate and multivariate Cox regression analyses were carried out on TCGA and E-MTAB-1980 cohort. In univariate Cox regression analyses, the risk score was a significantly prognostic factor for OS (TCGA cohort: HR =1.090, 95% CI =1.070–1.110, *p* < 0.001, **Figure 4A**; E-MTAB-1980 cohort: HR = 1.875, 95% CI= 1.357–2.590, *p* < 0.001, **Supplementary Figure 5A**). In the multivariate Cox regression analysis, the risk score remained as an independent prognostic variable for OS (TCGA cohort: HR =1.079, 95% CI = 1.058–1.101, *p* < 0.001, **Figure 4B**; E-MTAB-1980 cohort: HR = 1.773, 95% CI = 1.304–2.411, *p* < 0.001, **Supplementary Figure 5B**). A nomogram is a practical tool that can predict the likelihood of clinical events. Therefore, a nomogram model was constructed by integrating three variables according to the results of the multivariate Cox regression analyses in order to predict survival probability rates for individuals (**Figure 4C**). As can be seen in **Figures 4D–F**, the calibration curve illustrated that a good consistency was presented between the actual survival and nomogram predicted survival at 1, 3, and 5 years.

### Analysis expression of the genes from the signature in ccRCC tissues

The mRNA expression of the eight genes from the HMRGs prognostic signature was detected in five paired human ccRCC tissues and adjacent normal tissues. We found that the expression of IRF6, TEK, ABCB1, and COL4A5 was downregulated in ccRCC tissues compared to that in adjacent normal tissues, and the expression of PLCB2, TGFA, PLOD2, and TUBB6 was upregulated in ccRCC tissues compared to that in adjacent normal tissues (**Supplementary Figure 6**).

### Stratified analysis

**Figure 5:**
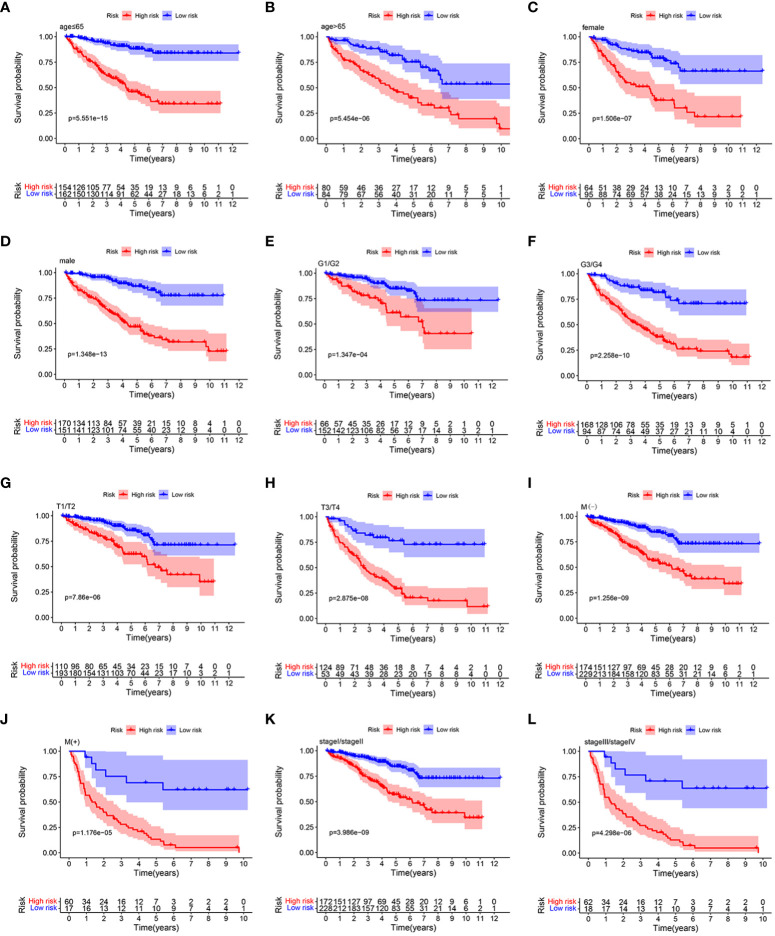
K–M survival analysis between the two risk groups in subgroups stratified by clinical characteristics, including age [ ≤ 65 years vs. >65 years] **(A, B)**, gender [female vs. male] **(C, D)**, tumor grade [G1/G2 vs. G3/G4] **(E, F)**, AJCC T stage [T1/T2 vs. T3/T4] **(G, H)**, AJCC M stage [M(–) vs. M(+)] **(I**, **J)**, and stages [stage I/stage III vs. stage III/stage IV] **(K, L)**, respectively.

Stratification analysis was conducted in order to further verify the performance of the HMRGs signature model to accurately and independently predict the clinical outcome of patients with ccRCC. Patients from the TCGA cohort were categorized into different subgroups based on age (≤65 vs. >65 years), gender (female vs. male), grade (G1/2 vs. G3/4), (AJCC) T stage (T1–2 vs. T3–4), (AJCC) M stage (M(−) vs. M(+)), and (AJCC) stage (stage I/II vs. stage III/IV). Then, K-M survival analysis was performed. We found that patients in the high-risk group consistently showed significantly poor prognosis in all subgroups, including age ≤65 years, age >65 years, female, male, grade G1/2, grade G3/4, T1–2, T3–4, M(−), M(+), stage I/II, and stage III/IV (**Figures 5A–L**). These results indicated the universal applicability of the HMRGs signature model for predicting prognostic in patients with ccRCC.

### Prognostic model risk score and clinical features

**Figure 6:**
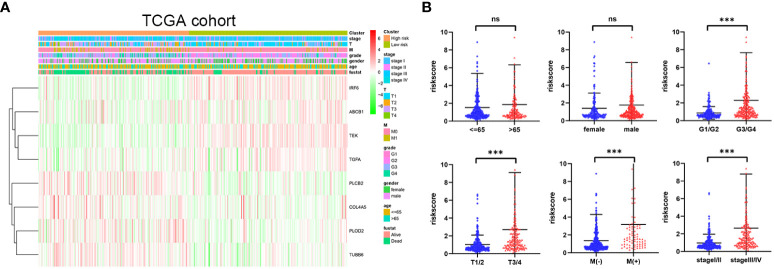
Correlation between risk score and clinicopathological parameters in the TCGA cohort **(A, B)**. *p-*values were shown as: ns, not significant; ***p < 0.001.

The distribution of risk score values was analyzed after stratification according to clinicopathological features for the purpose of further exploring the correlation between risk score and clinicopathological features. As can be seen in **Figures 6A, B**, from the TCGA cohort analysis results, it was indicated that patients with worse clinicopathological features, including high grade, advanced (AJCC) T stage, (AJCC) metastasis, and advanced (AJCC) TMN stage, had a significantly higher risk score. In addition, from the E-MTAB-1980 cohort analysis results, it was indicated that patients with advanced (AJCC) TMN stage or lymphatic metastasis were associated with an obviously higher risk score (**Supplementary Figures 7A, B**). In sum, as the risk score increased, the probability of developing advanced tumor gradually increased, indicating that the HMRGs signature may play an important role in the progression of ccRCC.

### Functional enrichment analyses in the TCGA cohort

**Figure 7:**
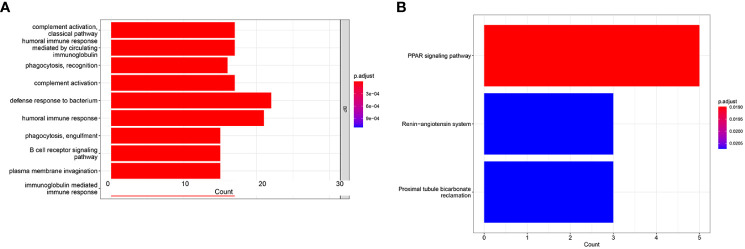
GO and KEGG analysis in the TCGA cohort. GO enrichment analysis **(A)**. KEGG enrichment analysis **(B)**.

To seek the potential biological functions and pathways of risk score-related genes, GO enrichment and KEGG pathway analyses were carried out based on the DEGs between the two risk groups. GO analysis revealed that DEGs mainly focused on several immune response processes, such as complement activation classical pathway, humoral immune responses mediated by circulating immunoglobulin, humoral immune responses, B-cell receptor signaling, and immunoglobulin-mediated immune responses (**Figure 7A**). Moreover, the KEGG analysis revealed that the PPAR signaling pathway was significantly enriched in those DEGs (**Figure 7B**).

### Relationship between risk score and immune infiltration landscape in the TCGA cohort

**Figure 8:**
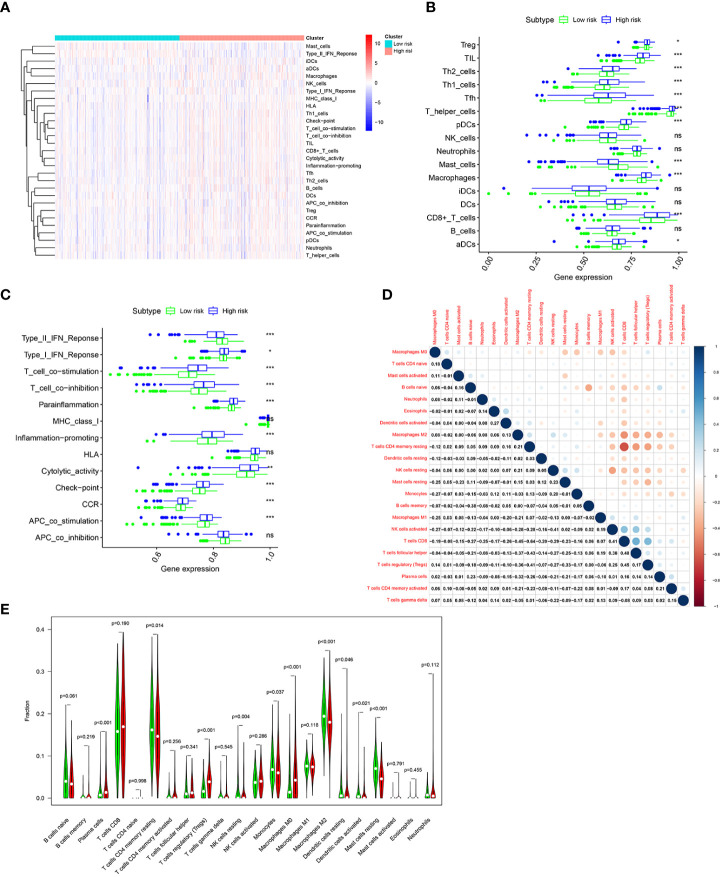
Immune infiltration pattern analysis based on risk characteristics in the TCGA cohort. **(A)** Relationship heatmap of the risk score and ssGSEA scores. Differential analysis of immune cells **(B)** and immune function **(C)** between the high-and low-risk groups based on ssGSEA. **(D)**. Correlation among 22 immune cell types based on the CIBERSORT algorithm. **(E)**. Differences in immune cell levels of the high- and low-risk groups based on the CIBERSORT algorithm. *p-*values were shown as: ns, not significant; *p < 0.05; **p < 0.01; ***p < 0.001.

To further investigate the difference of immune status between the two risk groups, ssGSEA algorithm was performed for the purpose of quantifying the enrichment scores of diverse immune cell subpopulations and immune-related functions between the two risk groups. As can be seen in **Figures 8A, B**, it was found that immune cell abundance, including Treg, TIL,Th2_cells, Th1_cell, Tfh,T_helper_cells, pDCs, macrophages, CD8+_T_cells, and aDCs, was significantly higher in the high-risk group. In addition, immune function scores, including Type_I_IFN_Response, T_cell_costimulation, T_cell_co-inhibition, Parainflammation, Inflammation-promoting, Cytolytic_activity, check-point, CCR, and APC_co_stimulation, were significantly stronger in the high-risk group (**Figure 8C**). The above results indicated that the immune response is more active in the high-risk group than in the low-risk group. Next, the CIBERSORT algorithm was applied for the purpose of comparing the proportion of 22 types of immune cells between the two risk groups. Correlations of these immune cells were depicted in **Figure 8D**. As shown in **Figure 8E**, plasma cells, regulatory T cells, and M0 macrophages were significantly higher in the high-risk group, while CD4 memory resting T cells, resting NK cells, monocytes, M2 macrophages, resting dendritic cells, activated dendritic cells, and resting mast cells were much higher in the low-risk group.

### Relationship between the prognostic signature and immunotherapy response

**Figure 9:**
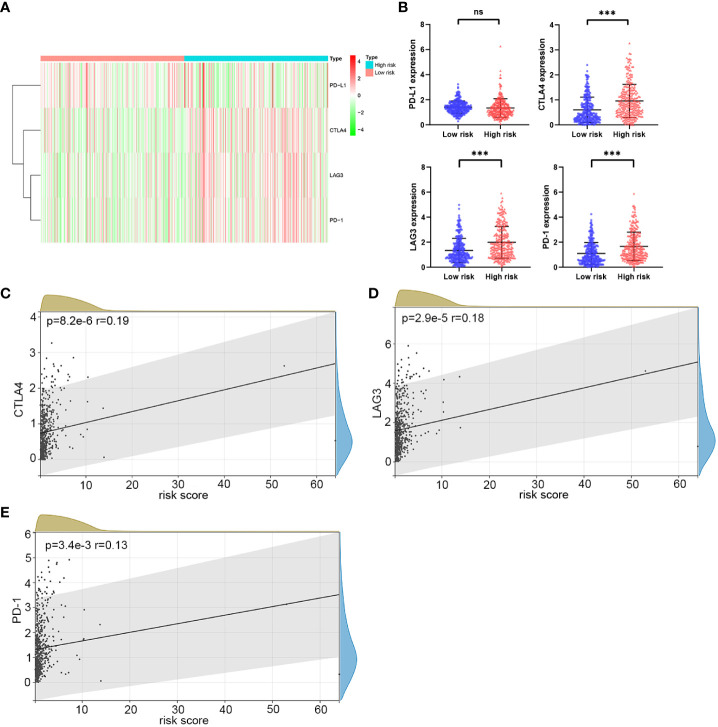
The correlation analysis of risk score and immune checkpoint molecules in the TCGA cohort. **(A, B)**. Heatmap of immune checkpoint molecule expression, including PD-1, CTLA4, LAG3, and PD-L1, based on risk characteristics. **(C–E)** The correlation between the risk score and the expression of immune checkpoints. *p*-values were shown as: ns, not significant; ***p < 0.001.

Immunotherapy is an important part of treatment for advanced ccRCC (3–5). Therefore, we sought to explore whether there is a relationship between risk scores and immunotherapy response. Common immune molecules, including PD-L1, CTLA4, LAG3, and PD-1 are important markers for personalized immunotherapy. It was clear that the expression of CTLA4, LAG3, and PD-1 was significantly higher in the high-risk group than in the low-risk group (**Figures 9A, B**). Moreover, the risk score was significantly positively correlated with the expression of CTLA4, LAG3, and PD-1 (**Figures 9C–E**). In short, patients in the high-risk group would benefit more from immunotherapy. Therefore, the HMRGs signature could predict the response to immunotherapy.

## Discussion

Hypoxia and metabolism are the important characteristics of TME and play a crucial role in the tumor occurrence, progression, angiogenesis, drug resistance, and immune response (12–14, 19–21). Considering the importance of the complex interaction between hypoxia and metabolism, it is appropriate to combine hypoxia and metabolism to construct a prognostic model with better accuracy for patients with ccRCC.

In this study, a signature was constructed based on the HMRGs, and the clinical prognostic value was confirmed. All patients were divided into either high-risk group or low-risk group based on the HMRGs-related risk score. Our results indicated that patients in the high-risk group had a significantly shorter OS time than their counterpart in the low-risk group. To further verify the prognostic value of the HMRGs signature model, ROC and independent prognostic analysis were performed, resulting in the signature model being capable of accurately and independently predicting ccRCC clinical prognoses. In addition, patients were assigned to different subgroups according to clinical pathological characteristic subtypes, especially when the clinicopathological factor typing of age, gender, tumor grade, (AJCC) T stage, (AJCC) M stage, and (AJCC) stage can consistently show significantly poor prognosis in the high-risk group, suggesting the universal applicability of the HMRGs signature model we constructed.

Our signature consisted of eight HMRGs signaling-related genes, namely, IRF6, TEK, PLCB2, ABCB1, TGFA, COL4A5, PLOD2, and TUBB6. IRF6 (interferon regulatory factor 6) belongs to the IRF family of transcription factors (22), which has been identified as a tumor suppressor in a variety of human cancers (23). Xu et al. found that IRF6 expression was downregulated in highly metastatic nasopharyngeal carcinoma. Overexpression of IRF6 suppressed nasopharyngeal carcinoma cell proliferation and growth *in vitro* (24). A recent study reported that IRF6 expression was downregulated in ccRCC, and overexpression of IRF6 suppressed the proliferation, invasion, and migration of ccRCC cells (25). TEK, also known as TIE-2, was a receptor tyrosine kinase, which plays a key role in vascular regeneration and stabilization (26). Chen et al. found that TEK expression was downregulated in ccRCC and that low TEK expression was associated with the poor survival of ccRCC patients. Knockdown of TEK facilitated proliferation and migration and suppressed apoptosis of ccRCC cells *in vitro* via promoting AKT phosphorylation (27). PLCB2 belongs to the phospholipase C beta (PLCB) gene family, which has been identified to play an important role in the development of various cancers (28, 29). Bertagnolo et al. demonstrated that PLCB2 was upregulated in breast cancer and abnormal increasing expression of PLCB2 was correlated with a poor clinical prognosis (30). ABCB1, also known as multidrug resistance protein 1 (MDR1), has been reported as a transport protein that functions to protect cells from the damage of xenobiotic and toxic substances, including anticancer drugs (31). Omori et al. found that elevated ABCB1 expression was associated with resistance to etoposide in small cell lung cancer (32). A recent review reported that the inhibition of ABCB1 could restore the drug sensitivity of the cancer cells toward chemotherapeutic drugs (33). TGFA belongs to the epidermal growth factor family, which could bind to EGFR to activate a series of signaling pathways involved in several biological processes, including cell proliferation, migration, differentiation, and energy metabolism (34, 35). COL4A5 (collagen type IV alpha 5 chain) is the important component of glomerular basement membrane, and it is well known that mutation or deficiency of COL4A5 was usually related with hereditary human diseases (36, 37). Recent evidence indicated that COL4A5 played an important role in tumor progression. Wu et al. found that COL4A5 expression was significantly upregulated in luminal breast cancer. Knockdown of COL4A5 significantly suppressed the growth of luminal-type breast cancer cells (38). Xiao et al. reported that COL4A5 facilitated proliferation and angiogenesis in lung cancer (39). PLOD2 belongs to the PLOD family, and it has been confirmed that PLOD2 mediated the formation of stabilized collagen cross-links, which played an important role in extracellular matrix (40). Zhen et al. found that PLOD2 expression was significantly high in osteosarcoma, which was associated with the poor survival of patients with osteosarcoma. Overexpression of PLOD2 facilitated osteosarcoma cell migration, invasion, and angiogenesis (41). Du et al. reported that PLOD2 expression was elevated in non-small-cell lung cancer and positively related to a poor prognosis of patients with non-small-cell lung cancer (42). Kurozumi et al. reported that PLOD2 was significantly upregulated in ccRCC and knockdown of PLOD2 significantly suppressed cell migration and invasion (43). TUBB6 (tubulin beta 6 class V) belongs to the β-tubulin family (44). A recent evidence indicated that high expression of TUBB6 was linked to a poor prognosis of patients with bladder cancer, and knockdown of TUBB6 significantly inhibited cell migration and invasion (45). Nami et al. found that aberrant expression of TUBB6 was involved in the potential mechanisms of taxane resistance in breast cancer (46).

Immune infiltration in the TME is a crucial factor affecting tumor progression and response to immunotherapy. GO analysis revealed differing immune-related pathway activities between high-risk and low-risk groups, including complement activation classical pathway, humoral immune responses mediated by circulating immunoglobulin, humoral immune responses, B-cell receptor signaling, and immunoglobulin mediated immune responses. The ssGSEA algorithm showed differing infiltrating scores of immune-cell and immunity-related pathways between the two groups. The results of the CIBERSORT algorithm indicated that patients in the high-risk group had significantly increased infiltration of plasma cells, regulatory T cells, and M0 macrophages, while patients in the low-risk group had significantly increased infiltration of CD4 memory resting T cells, resting NK cells, monocytes, M2 macrophages, resting dendritic cells, activated dendritic cells, and resting mast cells. Tregs could induce immune tolerance and promote immune escape and cancer metastasis (47, 48). Previous studies have verified that T cells’ regulatory infiltration was related to the poor survival of ccRCC patients (49, 50). Pan et al. reported that increased resting mast cells’ density was linked to a favorable prognosis in ccRCC patients (51). Zhang et al. found that ccRCC patients with low risk had increased abundance of CD4 memory resting T cells, resting NK cells, monocytes, and M2 macrophages (52).

Immunotherapy is transformative in treating advanced ccRCC (4, 6). However, there are still a significant number of patients with no response or resistance to immunotherapy (53). Constructing predictive biomarkers for immunotherapy could assist in screening the appropriate patients to achieve precise treatment. Our study used the HMRGs signature to evaluate the response to immunotherapy between the two risk groups. We found that the expression of CTLA4, LAG3, and PD-1 were significantly higher in the high-risk group, suggesting that patients in the high-risk group could better respond to immunotherapy. Moreover, the risk score was significantly positively correlated with the expression of CTLA4, LAG3, and PD-1. These results consistently show that high-risk patients would benefit more from immunotherapy.

However, several limitations existed in the present study. First, all the conclusions in our study were obtained from bioinformatic analysis, and a prospective multiple clinical trial validation was needed to develop a higher evidence level of findings. Second, further experimental studies are needed to explore the specific function and mechanisms of these genes in relation to the progression of ccRCC.

## Conclusion

The HMRGs prognostic signature was established based on the integrated analysis of hypoxia- and metabolism-related genes, which was confirmed to be a reliable predictor for OS in ccRCC. Moreover, this signature was correlated with the expression of common immune checkpoints, which could assist in guiding immunotherapy decisions in order to achieve precise treatment.

## Data availability statement

The original contributions presented in the study are included in the article/**Supplementary material**. Further inquiries can be directed to the corresponding author.

## Author contributions

LL and XW were responsible for the study design and writing. WX and BG were mainly responsible for data analysis. BF, WC, and LZ were mainly responsible for data collection. LL and XW were responsible for manuscript review and providing constructive comments. All authors contributed to the article and approved the submitted version.

## Supplementary material

Supplementary Figure 1 The flowchart for the process of constructing the HMRG prognostic signature.

Supplementary Figure 2 **(A–H)** Analysis expression of the prognostic signature genes (IRF6, TEK, PLCB2, ABCB1, TGFA, COL4A5, PLOD2, and TUBB6) in ccRCC tissues compared with normal tissues in the TCGA data.

Supplementary Figure 3 **(A–H)** The survival plot for the signature genes (IRF6, TEK, PLCB2, ABCB1, TGFA, COL4A5, PLOD2, and TUBB6) in the TCGA data.

Supplementary Figure 4 Validation of the HMRG prognostic signature in the E-MTAB-1980 cohort. **(A)** Distribution of patients’ risk score. **(B)** Survival status. **(C)** Expression of eight signature genes. **(D)** K-M curves for the OS. **(E)** ROC curve for 1, 3, and 5 years.

Supplementary Figure 5 Univariate **(A)** and multivariate Cox regression **(B)** were performed in the E-MTAB-1980 cohort.

Supplementary Figure 6 Analysis expression of the prognostic signature genes (IRF6, TEK, PLCB2, ABCB1, TGFA, COL4A5, PLOD2, and TUBB6) in five paired human ccRCC tissues and adjacent non-tumorous tissues.

Supplementary Figure 7 Correlation between risk score and clinicopathological parameters in the E-MTAB-1980 cohort **(A, B)**. *p-*values were shown as: ns, not significant; **p* < 0.05; ***p* < 0.01.
