# Supplementary figures and images for: Development and validation of a combined hypoxia- and metabolism-related prognostic signature to predict clinical prognosis and immunotherapy responses in clear cell renal cell carcinoma
Source: Front Oncol. 2023 Nov 10;13:1162846. doi: 10.3389/fonc.2023.1162846 (PMC10667439; doi:10.3389/fonc.2023.1162846)

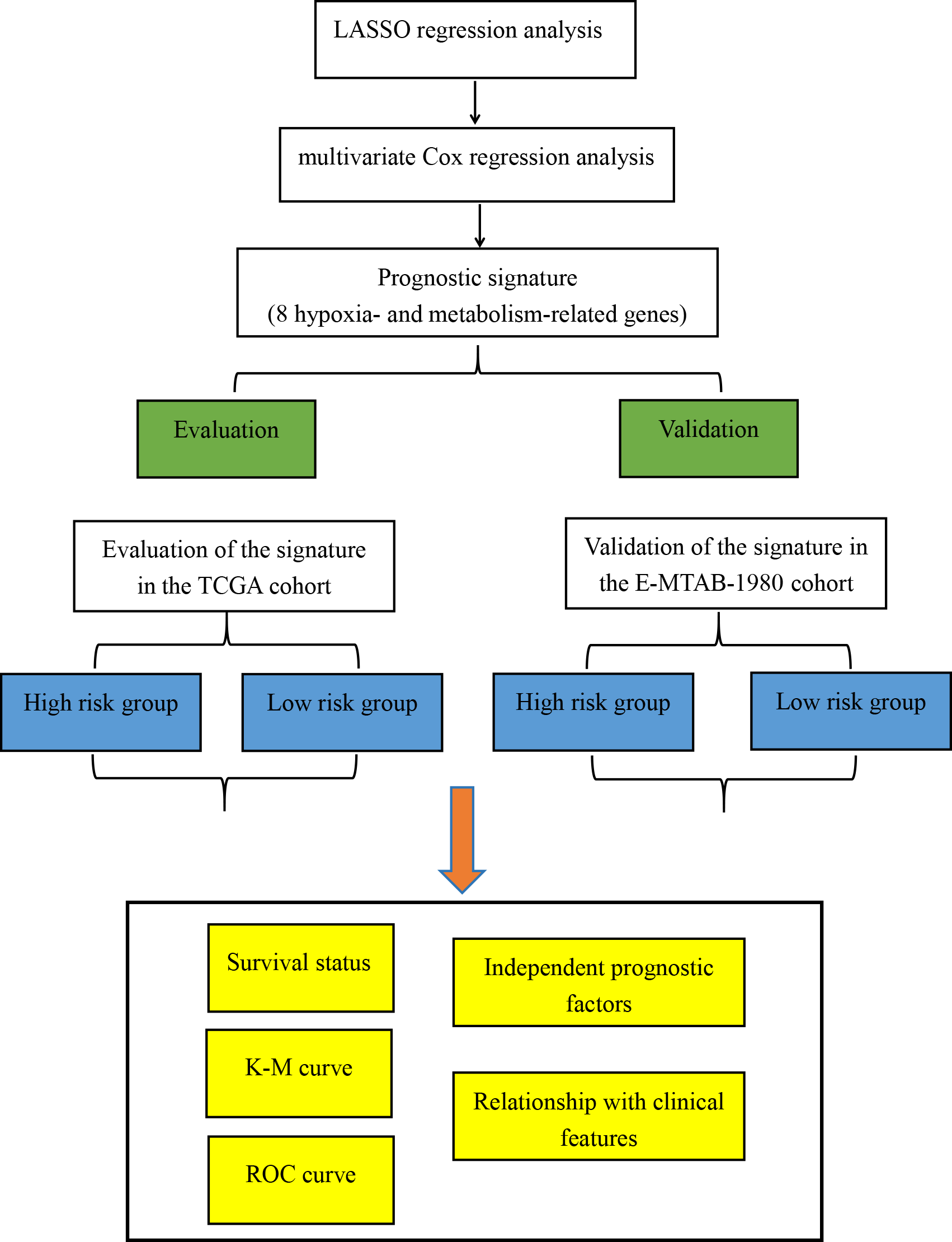

Supplement: Supplementary Figure 1 — The flowchart for the process of constructing the HMRG prognostic signature. [file Image_1.tif]

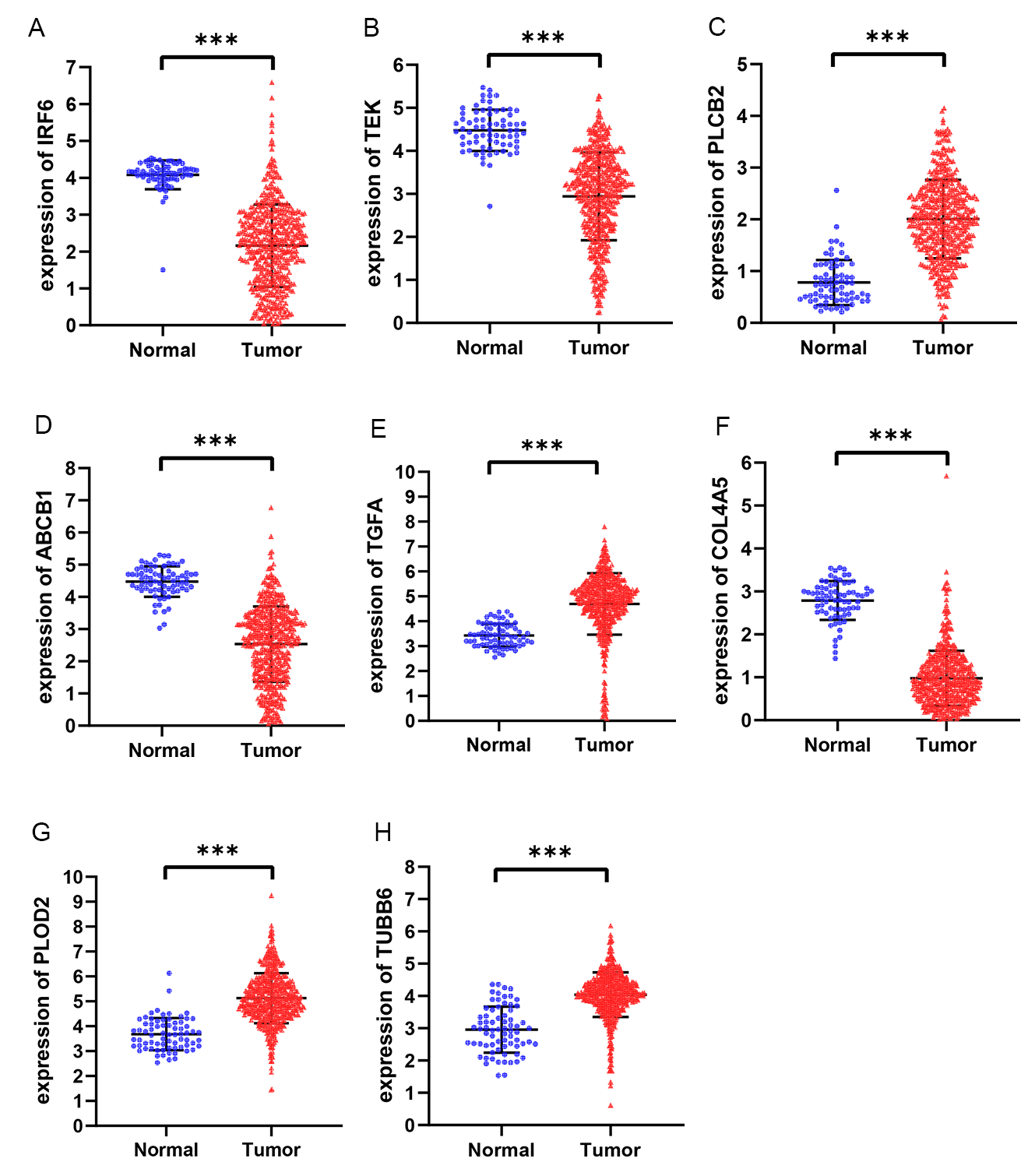

Supplement: Supplementary Figure 2 — (A–H) Analysis expression of the prognostic signature genes (IRF6, TEK, PLCB2, ABCB1, TGFA, COL4A5, PLOD2, and TUBB6) in ccRCC tissues compared with normal tissues in the TCGA data. [file Image_2.tif]

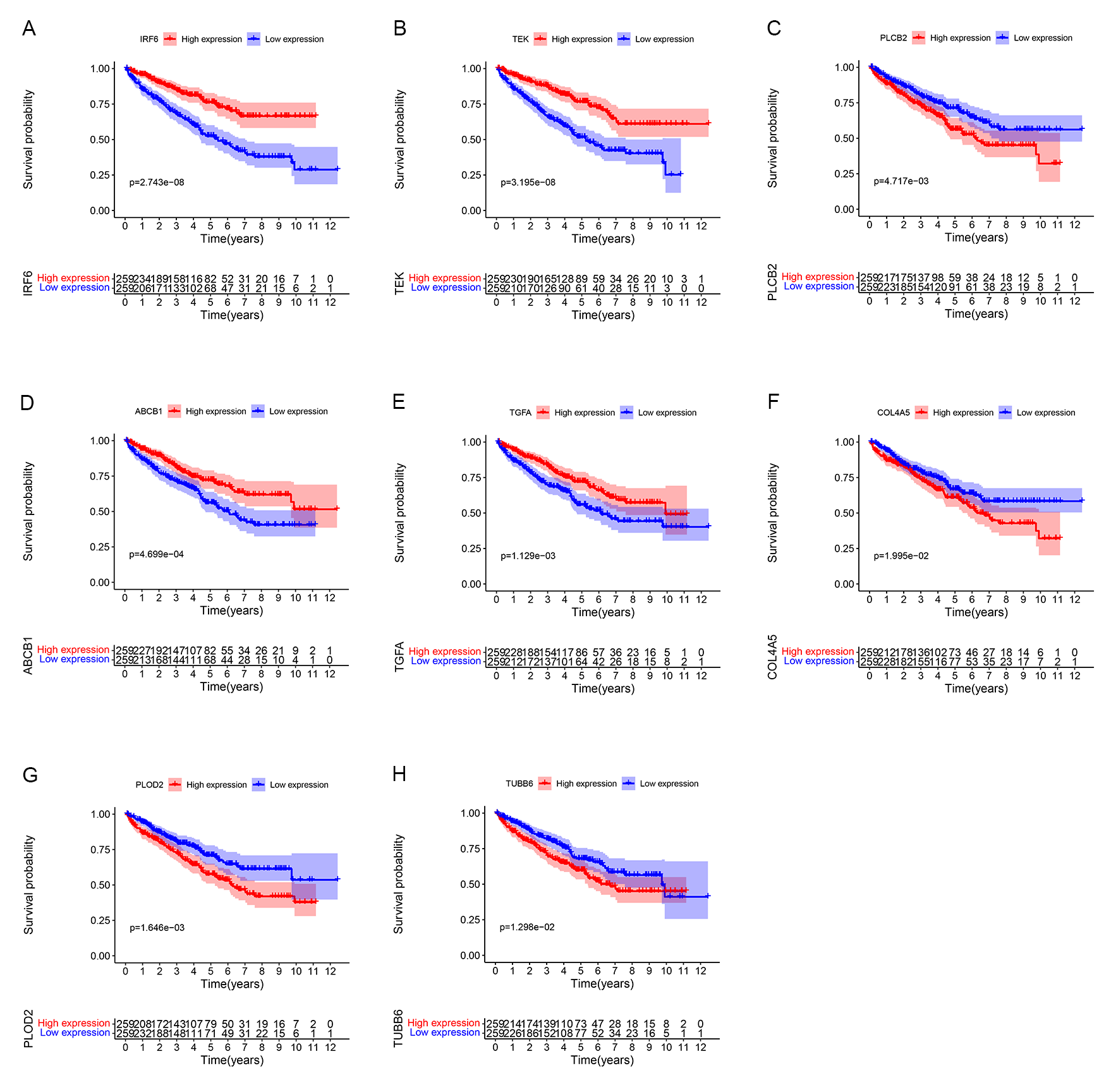

Supplement: Supplementary Figure 3 — (A–H) The survival plot for the signature genes (IRF6, TEK, PLCB2, ABCB1, TGFA, COL4A5, PLOD2, and TUBB6) in the TCGA data. [file Image_3.tif]

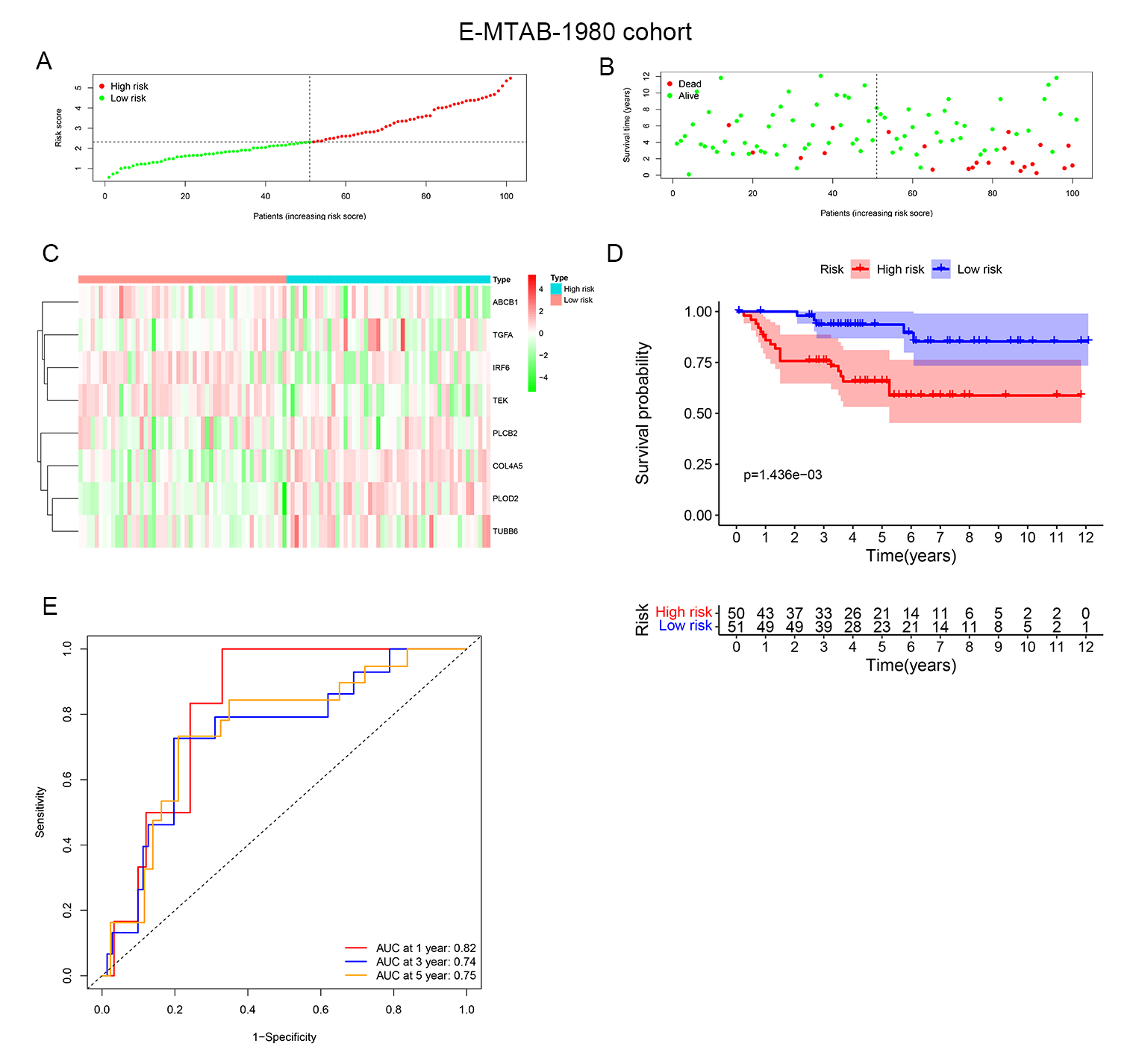

Supplement: Supplementary Figure 4 — Validation of the HMRG prognostic signature in the E-MTAB-1980 cohort. (A) Distribution of patients’ risk score. (B) Survival status. (C) Expression of eight signature genes. (D) K-M curves for the OS. (E) ROC curve for 1, 3, and 5 years. [file Image_4.tif]

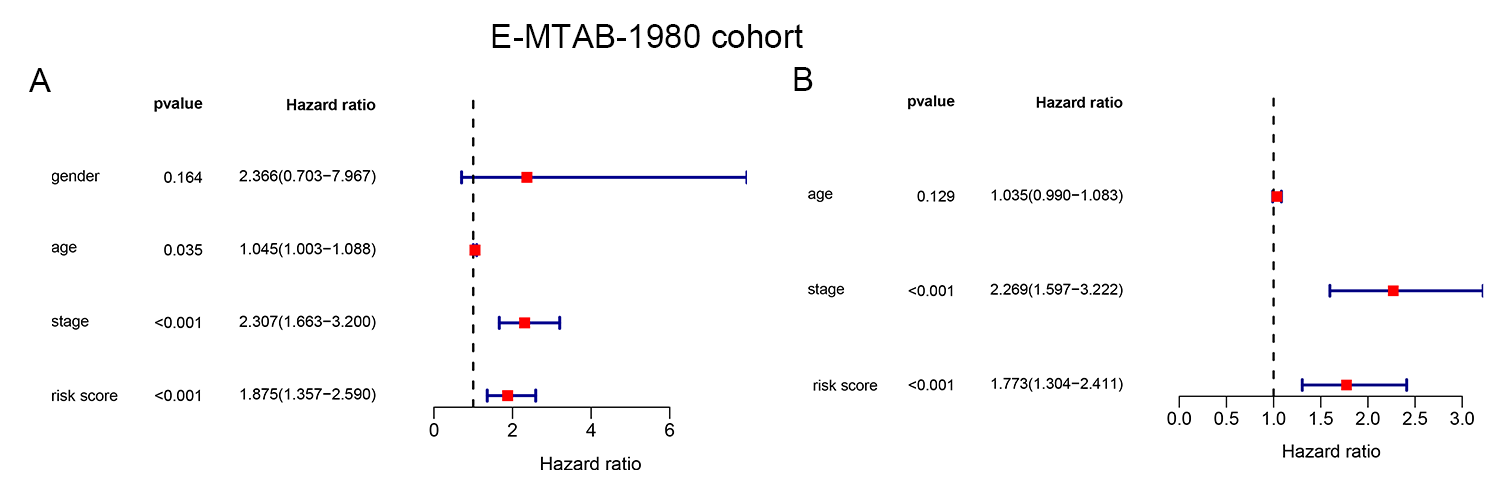

Supplement: Supplementary Figure 5 — Univariate (A) and multivariate Cox regression (B) were performed in the E-MTAB-1980 cohort. [file Image_5.tif]

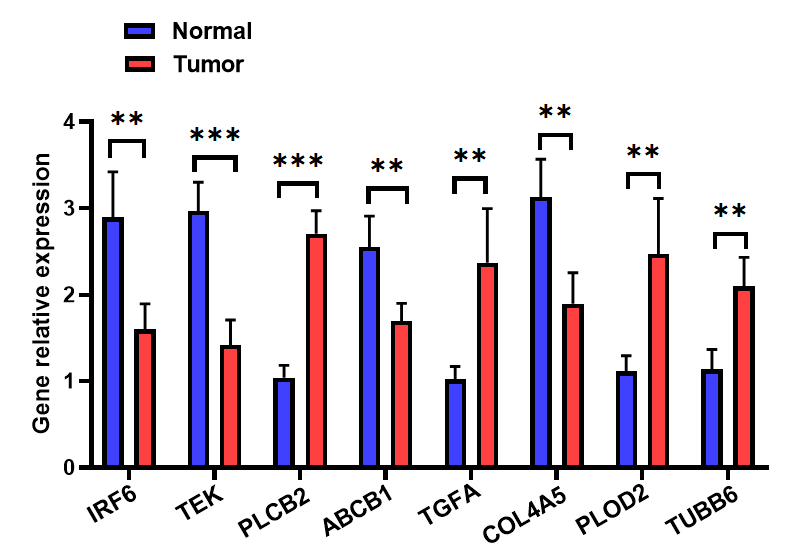

Supplement: Supplementary Figure 6 — Analysis expression of the prognostic signature genes (IRF6, TEK, PLCB2, ABCB1, TGFA, COL4A5, PLOD2, and TUBB6) in five paired human ccRCC tissues and adjacent non-tumorous tissues. [file Image_6.tif]

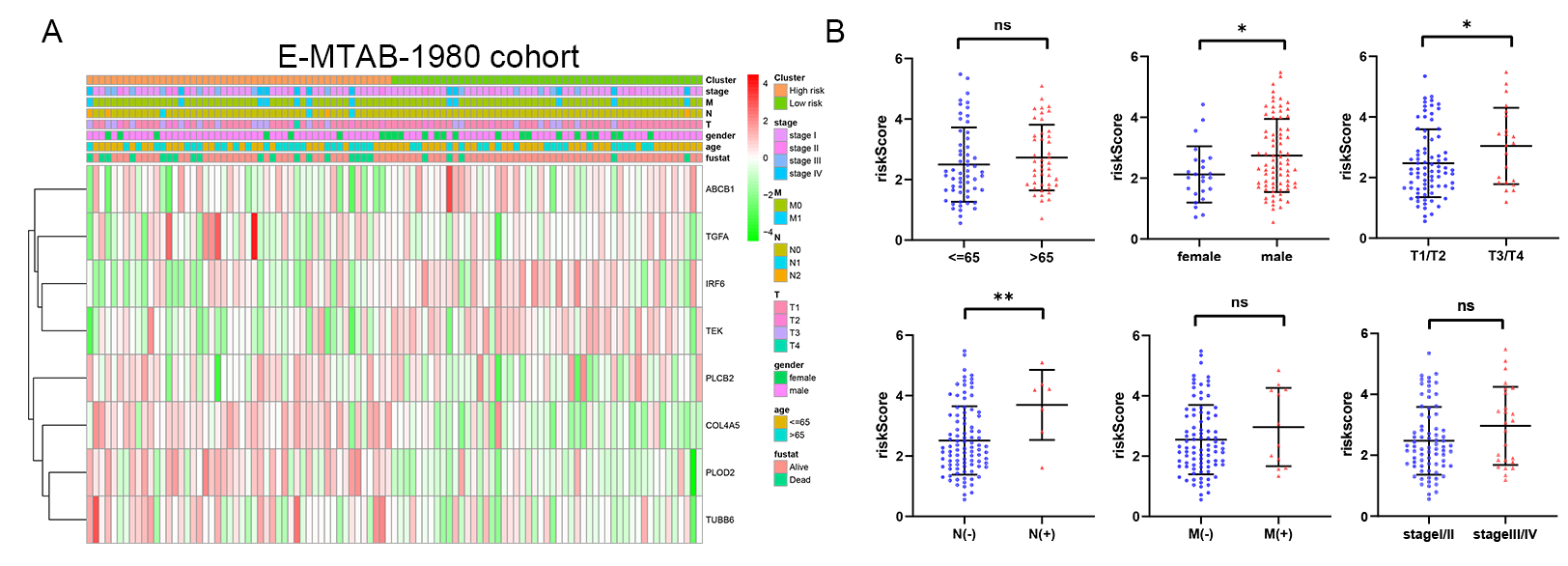

Supplement: Supplementary Figure 7 — Correlation between risk score and clinicopathological parameters in the E-MTAB-1980 cohort (A, B). p-values were shown as: ns, not significant; *p < 0.05; **p < 0.01. [file Image_7.tif]
