# Supplementary material for: Development and validation of a combined hypoxia- and metabolism-related prognostic signature to predict clinical prognosis and immunotherapy responses in clear cell renal cell carcinoma
Source: Front Oncol. 2023 Nov 10;13:1162846. doi: 10.3389/fonc.2023.1162846 (PMC10667439; doi:10.3389/fonc.2023.1162846)
Supplement: Supplementary file 9 [file Table_2.doc]

| Primer | Sequence |
| --- | --- |
| IRF6 Forward Primer | CCCCAGGCACCTATACAGC |
| IRF6 Reverse Primer | TCCTTCCCACGGTACTGAAAC |
| TEK Forward Primer | TTAGCCAGCTTAGTTCTCTGTGG |
| TEK Reverse Primer | AGCATCAGATACAAGAGGTAGGG |
| PLCB2 Forward Primer | ATCCGGGATACTCGCTTTGG |
| PLCB2 Reverse Primer | CACCACCGTGAGTGTCTTCAG |
| ABCB1 Forward Primer | TTGCTGCTTACATTCAGGTTTCA |
| ABCB1 Reverse Primer | AGCCTATCTCCTGTCGCATTA |
| TGFA Forward Primer | AGGTCCGAAAACACTGTGAGT |
| TGFA Reverse Primer | AGCAAGCGGTTCTTCCCTTC |
| COL4A5 Forward Primer | TGGACAGGATGGATTGCCAG |
| COL4A5 Reverse Primer | GGGGACCTCTTTCACCCTTAAAA |
| PLOD2 Forward Primer | CATGGACACAGGATAATGGCTG |
| PLOD2 Forward Primer | AGGGGTTGGTTGCTCAATAAAAA |
| TUBB6 Forward Primer | TGGTGGACTTAGAGCCAGG |
| TUBB6 Reverse Primer | CCCTTTCGCCCAGTTGTTC |
| GAPDH Forward Primer | GGAGCGAGATCCCTCCAAAAT |
| GAPDH Reverse Primer | GGCTGTTGTCATACTTCTCATGG |

**Supplementary Table 2**.The sequence of primers in our study.
